# In vitro growth and characterization of ‘Candidatus Sarcina troglodytae’ isolated from the brain of a chimpanzee in Sierra Leone

**DOI:** 10.1099/acmi.0.001186.v4

**Published:** 2026-10-07

**Authors:** Emily Dunay, Gregory A. Barrett-Wilt, Leah A. Owens, Ismail Hirji, Tony L. Goldberg, Johanna R. Elfenbein

**Affiliations:** 1 Department of Pathobiological Sciences, School of Veterinary Medicine, University of Wisconsin-Madison, Madison, WI, USA; 2 Biotechnology Center, University of Wisconsin-Madison, Madison, WI, USA; 3 Tacugama Chimpanzee Sanctuary, Freetown, Sierra Leone

**Keywords:** anaerobe, chimpanzee, endospore, pan troglodytes, *Sarcina*

## Abstract

‘*Candidatus* Sarcina troglodytae’ (‘*Ca*. S. troglodytae’), a proposed bacterial species in the family *Clostridiaceae*, has been epidemiologically associated with a lethal disease in sanctuary chimpanzees in Sierra Leone, epizootic neurologic and gastroenteric syndrome (ENGS). As sarcinae are known to be difficult to culture, a lack of viable *in vitro* growth conditions has limited further characterization of this bacterium. Such studies are critical to elucidating the relationship between ‘*Ca*. S. troglodytae’ and ENGS. Here, we isolate a Gram-stain-positive, endospore-forming coccus and anaerobic bacterial strain, designated JB3^T^, from brain tissue of a western chimpanzee (*Pan troglodytes verus*) using a reinforced clostridial medium. We describe the growth of strain JB3^T^ at a pH range of 6 to 9 (optimum 6) at 37 °C in an atmosphere composed of 5% H_2_, 5% CO_2_ and 90% N_2_ and demonstrate endospore production under alkaline conditions, suggesting the possibility of an environmental reservoir. We identify ethanol as the primary non-gaseous fermentation byproduct which we hypothesize to have relevance for ENGS. Antibiotic susceptibility testing shows susceptibility to common antimicrobial agents with notable resistance to ceftriaxone. By establishing *in vitro* conditions for the viable culture, propagation and storage of ‘*Ca*. S. troglodytae’, this study lays the groundwork for future infection studies to test hypotheses about pathogenic mechanisms and prevention/treatment measures for ENGS.

## Data Summary

The 16S rRNA gene sequence of the ‘*Ca.* S. troglodytae’ type strain JB3^T^ is available in GenBank under the accession number PQ137441. The type strain JB3^T^ is publicly available in the American Type Culture Collection (=ATCC TSD-457^T^).

## Introduction

Bacteria in the genus *Sarcina* (family *Clostridiaceae*) are Gram-stain-positive, tetrad packet-forming cocci and carbohydrate-fermenting anaerobes [1]. The genus was first described in 1842 by Goodsir when the type species *Sarcina ventriculi* was observed in the stomach contents of a human with recurrent vomiting [2]. Subsequently, *Sarcina maxima*, the only other recognized species in this genus, was observed by Linder in spontaneously souring flour paste in 1888 [1]. Based on phylogenetics using the 16S rRNA gene, *Sarcina* spp. cluster within group I of the genus *Clostridium*; however, the genus name *Sarcina* takes precedence, having been proposed prior to *Clostridium* [3]. *Sarcina* bacteria have been characterized by their ability to survive a wide pH range (1–9.8), to form endospores at alkaline pH and to be difficult to culture due to rapid loss of viability *in vitro* [1, 4].

Since their initial discovery, sarcinae have been identified in healthy humans, domestic animals and wildlife, as well as the environment; however, these bacteria have also been implicated in disease [5–8]. *S. ventriculi* has been associated with gastrointestinal disease in humans, particularly in individuals with delayed gastric emptying, which may facilitate bacterial overgrowth and opportunistic disease, although such cases have rarely been fatal [9]. In contrast, sarcinae have also been associated with often fatal abomasal bloat in young ruminants [10, 11] and gastric dilation in horses [12], dogs [12] and macaques [13]. To our knowledge, *S. maxima* has not been associated with disease. Nevertheless, the lack of species-specific diagnostic assays and difficulty culturing sarcinae have often prohibited species identification in clinical cases, such that the true diversity of sarcinae associated with clinical disease remains obscure [14, 15].

Previously, we investigated a uniformly lethal disease of unknown aetiology affecting a population of wild-born, sanctuary-housed western chimpanzees (*Pan troglodytes verus*) in Sierra Leone [15]. Using a case-control study design, we revealed a genetically distinct *Sarcina* bacterium, ‘*Candidatus* Sarcina troglodytae’ (‘*Ca*. S. troglodytae’) [16], to be associated with the disease, which was termed epizootic neurologic and gastroenteric syndrome (ENGS). We then conducted an epidemiological study to determine the distribution and prevalence of ‘*Ca*. S. troglodytae’ and related bacteria in chimpanzee populations and their environments across Africa, identifying several demographic and ecological risk factors for the presence of ‘*Ca*. S. troglodytae’ [14]. We previously performed phylogenetic analysis of the 16S rRNA gene and whole-genome sequencing on an isolate from the brain of an ENGS affected chimpanzee (strain JB2, GenBank acc. no. CP051754); however, viable *in vitro* growth conditions were not established at that time [15]. Here, we describe such conditions and confirm isolate identity by independent sequencing of the nearly complete 16S rRNA gene of strain JB3^T^. Through further *in vitro* experiments, we elucidate fermentation byproducts with potential clinical relevance, demonstrate the influence of pH on growth and sporulation and assess antibiotic susceptibility.

## Methods

### Isolation

Strain JB3^T^ was isolated from brain tissue of a wild-born, sanctuary-housed western chimpanzee (*Pan troglodytes verus*) at Tacugama Chimpanzee Sanctuary (TCS) in Sierra Leone (8.41710, –13.20663). This individual, a 13-year-old male, died suddenly after an episode of severe weakness and collapse on 27 November 2015, at which time brain tissue was collected as part of a routine necropsy, fresh frozen at −80 °C, shipped to the USA on dry ice and frozen at −80 °C. The post-mortem interval was ~4–8 h, and there was no visible autolysis on histopathology. The same brain tissue sample was previously used to isolate strain JB2 (GenBank acc. no. CP051754) for whole-genome sequencing in our prior study [15].

All isolation steps were performed within an anaerobic chamber with an atmosphere composed of 5% H_2_, 5% CO_2_ and 90% N_2_ (Shel Lab; Bactron EZ). A deep-cut section of frozen brain tissue was collected with a 6 mm biopsy punch, placed in a sterile Petri dish and minced with two sterile blades until homogenized. The minced tissue was plated with a sterile polyester swab (Fisher Scientific) onto reinforced clostridial (RC) medium plates (Thermo Scientific CM0149B RC Medium) supplemented with 10 g l^−1^ of Difco agar (Becton Dickinson) and incubated at 37 °C for 24–48 h. Resulting colonies were streaked and incubated for 24 h. *Sarcina* morphology was confirmed by microscopy, and the identity of the bacterium was confirmed by species-specific PCR, as previously described [14]. A single colony was then inoculated into RC broth (made in-house; Table S1, available in the online Supplementary Material) and incubated at 37 °C for 24 h. Glycerol stocks were made by the addition of a sterile equal volume of 20% glycerol to liquid culture (final concentration of 10% glycerol), incubated at room temperature for 15 min and frozen at −80 °C.

### 16S rRNA sequencing

DNA extraction from a colony of strain JB3^T^ was performed using the DNeasy Blood and Tissue Kit (Qiagen) according to the manufacturer’s instructions for cultured cells. PCR was performed in a 25 µl reaction volume containing 0.2 µM of each primer, 12.5 µl of 2×HotStar Master Mix (Qiagen), 9.5 µl of PCR-grade water and 2 ul of template DNA using universal 16S rRNA gene primers fD1 and rP2 [17, 18] under specified cycling conditions (95 °C for 15 min; 30 cycles of 94 °C for 45 s, 50 °C for 45 s, 72 °C for 90 s; and 72 °C for 10 min) on a T100 thermal cycler (Bio-Rad). The PCR product was visualized on a 1% agarose gel stained with ethidium bromide using a UV light GelDoc XR imager (Bio-Rad). The crude PCR product was purified using ExoSAP enzymatic cleanup and Sanger sequenced on an ABI 3730xl instrument (Applied Biosystems) at Functional Biosciences (Madison, WI, USA) using universal 16S rRNA gene primers fD1/8F and rP2 [18], 518R [19], 806R [20], 1100R [21], 515F [22] and 1100F [23]. Chromatograms were assembled using Sequencher 4.10.1 (Gene Codes).

### Fermentation byproducts

Gas production was assessed using the Durham tube test [24]. Specifically, a 6×50 mm glass culture tube (Kimble) was placed in an inverted position within a standard size glass culture tube containing RC broth prior to autoclaving. After inoculation with strain JB3^T^, the culture was incubated anaerobically at 37 °C for 24 h and then visually examined for gas production.

To characterize non-gaseous fermentation byproducts (e.g. ethanol and short-chain fatty acids), 24-h liquid culture supernatants of *S. maxima* (ATCC 33910), *S. ventriculi* (ATCC 29069) and strain JB3^T^ were analysed using an Agilent 7890/5977 Gas Chromatograph/Mass Spectrometer instrument, as previously described [25], at the University of Wisconsin-Madison Mass Spectrometry Facility, using uninoculated RC broth as a baseline reference.

### Influence of pH on growth and sporulation

The pH growth range of JB3^T^ was assessed from 3 to 10 in increments of 1–2 pH units. Growth curves were generated at pH 4, 6, 8 and 9. The media pH was adjusted sterilely using either hydrochloric acid 1 N solution or sodium hydroxide solution 10 N (Fisher Chemical) and an AB15 pH meter (Fisher Scientific). RC broth (9 ml) was inoculated with a 1 ml glycerol stock of strain JB3^T^ and incubated at 37 °C. After 24 h, 5 ml of the starting culture was diluted into 250 ml of RC broth and incubated at 37 °C for the duration of the experiment. Aliquots were taken at 0, 2, 4, 6, 8, 10, 12 and 24 h, serially diluted in RC broth and plated to quantify c.f.u. per millilitre (c.f.u. ml^−1^). Plates were incubated for 24 h at 37 °C and then the number of c.f.u. quantified. Data were assessed for normality using Shapiro-Wilk tests and analysed using Kruskal-Wallis tests with post hoc Wilcoxon rank-sum tests and Benjamini–Hochberg adjustment for multiple comparisons in GraphPad Prism 10. Endospores were visualized using malachite green stain according to the manufacturer’s instructions (Ward’s Science).

### Antibiotic susceptibility

Antibiotic susceptibility testing was performed using the disc diffusion method as previously described for isolates of *S. ventriculi* from faeces of healthy monkeys [17]. Eleven antibiotics were selected for testing based on common use and availability at TCS. Susceptibility testing was conducted using RC medium supplemented with 10 g l^−1^ of Difco agar. Freshly grown JB3^T^ liquid culture was spread over the agar plate surface using a sterile polyester swab. Antibiotic discs (BBL Sensi-Disc, Becton Dickinson) were placed onto the agar plate surface using sterile tweezers (TWD Scientific). For metronidazole, antibiotic discs were made in-house by creating a 0.5 mg ml^−1^ solution of metronidazole (DOT Scientific Inc.) in PBS and transferring 10 µl (5 µg of metronidazole) of the solution onto a blank disc (Thermo Scientific), which was then dried at room temperature. A blank disc was used as a negative control, and all antibiotics were tested in triplicate. Plates were incubated anaerobically for 24 h at 37 °C. Results were interpreted as susceptible, moderately susceptible or resistant, as previously described [17].

## Results and discussion

### Growth and morphology

Bacterial cells are Gram-stain-positive, anaerobic, tetrad packet-forming cocci, nonmotile and ~4–4.5 µm in diameter. Off-white in colour, opaque, circular colonies are formed after 24 h of anaerobic growth on RC plates at 37 °C. Colonies are smooth and glistening, raised, range in diameter from <1 to 4 mm and have entire margins. Strain JB3^T^ survived repeated passages every 24–48 h on RC plates and in RC broth. Following American Type Culture Collection (ATCC) guidelines for recovery from cryopreservation, glycerol stocks of strain JB3^T^ were repeatedly recoverable. In our published work [15], we demonstrated that strain JB2 is Gram-positive, and methylene blue staining showed a cellulose-containing cell wall [15]. The latter is exhibited by *S. ventriculi* but not *S. maxima* (Table 1). The cellular diameter of strain JB2 (mean 4.29 µm) falls outside the published range for *S. ventriculi* (1.8–2.4 µm) and *S. maxima* (2–3 µm) [15, 26]. Lastly, strain JB2 displays a flattened cellular morphology and large packet size similar to *S. ventriculi* [15, 27].

**Table 1.** Differential characteristics between ‘*Ca*. S. troglodytae’ and related species of the genus *Sarcina* Table adapted from reference [26].

Characteristic	‘*Ca.* S. troglodytae’	*S. ventriculi*	*S. maxima*
Cellulose-containing cell wall	Yes [15]	Yes	No
Cell diameter (µm)	4.29 (mean) [15]	2.83 (mean) [15] 1.8–2.4	2–3
Cell and packet morphology	Large packet size, flattened [15]	Packets contain more cells, irregular, flattened	Uniform
Spore formation at alkaline pH	Yes	Yes	Yes
pH range	6.0–9.0*	1.0–9.8	1.0–9.8
Primary non-gas fermentation byproduct	Ethanol	Ethanol	Butyric acid
Associated with disease	Yes	Yes	No

* At pH 4, cell density was observed to increase in liquid culture, but viable cultures were not retrieved.

Rapid loss of viability in culture has been reported for *S. ventriculi* and *S. maxima*, the reasons for which are unknown [1, 26]. Similarly, we found that liquid cultures of strain JB3^T^ were only viable for up to 48 h at 37 °C (Table S2). The genus *Sarcina* has also been described as relatively aerotolerant [26]. Likewise, when 24-h liquid cultures of strain JB3^T^ were exposed to oxygen for 3 h at room temperature and then returned to the anaerobic environment, plating yielded typical growth (Table S2). Differential characteristics of *Sarcina* spp. are summarized in Table 1.

### 16S rRNA gene sequencing

Independent 16S sequencing of strain JB3^T^ generated a nearly complete 16S rRNA gene (1,451 bp, GenBank acc. no. PQ137441) which shares 100% nt sequence identity to strain JB2 (GenBank acc. no. CP051754) from which the whole genome was previously sequenced [15], confirming isolate identity as ‘*Ca*. S. troglodytae’. We note that additional analyses such as whole-genome sequencing of strain JB3^T^ would be required to verify that these two isolates are indeed clones. We also note that our prior work, in combination with *in vitro* characterization in the present study, demonstrates differences in bacterial morphology, growth characteristics and genetic sequences that distinguish ‘*Ca*. S. troglodytae’ from other members of the genus *Sarcina*, including *S. ventriculi*, even though ‘*Ca*. S. troglodytae’ is closely related to other members of the genus *Sarcina* based on sequence analysis of barcoding genes [14, 15]. Nevertheless, we cannot rule out the possibility that strains JB2 and JB3^T^ represent a distinct ecotype of *S. ventriculi*, rather than a new species. This uncertainty highlights that the genus *Sarcina* is understudied and that future research to clarify relationships among members of genus *Sarcina* would be of great benefit to the field of bacterial microbiology.

### Fermentation byproducts

Bubbling of liquid cultures of strain JB3^T^ was observed, as described for other sarcinae [26], and gas production was observed using the Durham tube test [24]. After inoculation with strain JB3^T^ and anaerobic growth at 37 °C for 24 h, a bubble was observed trapped inside the inverted tube, indicating gas production. Most gas-producing microbes in phylum *Firmicutes*, including *S. maxima*, *S. ventriculi* and members of the closely related genus *Clostridium*, are known to produce CO_2_ and H_2_ as a result of carbohydrate fermentation [1, 28].

Of the analytes included in the assay (i.e. acetic, butyric, propionic, isobutyric, valerenic and succinic acids and ethanol), strain JB3^T^ predominantly produced ethanol, as did *S. ventriculi* (Table S3). As previously reported, *S. maxima* predominantly produced butyric acid, and ethanol was not detected [1]. Additionally, all three organisms produced acetic acid, which has been previously described for *S. ventriculi* and *S. maxima* [1]. Fermentation byproducts including ethanol and butyric acid are known to have pathophysiological relevance in the case of, for example, auto-brewery syndrome caused by various fungi and bacteria [29], in which excessive ethanol production results in signs and symptoms consistent with exogenous alcohol intoxication and alterations to *Clostridioides difficile* virulence [30], respectively. Given the isolation of strain JB3^T^ from the brain tissue of an individual that succumbed to ENGS and neurologic signs characterizing the syndrome (i.e., weakness, ataxia and seizures), we hypothesize that the production of ethanol as a result of carbohydrate fermentation may have relevance for the pathophysiology of disease. It has been speculated that in *Sarcina*-associated disease, metabolism of ethanol in the gastrointestinal tract results in acetaldehyde accumulation locally, causing mucosal injuries similar to those observed in humans with alcohol poisoning [10, 13]. These ethanol-induced gastric mucosal lesions damage the once-protective mucosal barrier, providing conditions for bacteria to spread from the stomach to the abdominal cavity, causing peritonitis that can progress to sepsis if untreated.

### Influence of pH on growth and sporulation

Growth was observed within the pH range of 4 to 9. At pH 8 and 9, endospore formation was observed. Endospores were circular and reached up to 30 µm in diameter (Fig. 1). Endospore formation by *S. maxima* and *S. ventriculi* at alkaline pH has been reported [4, 26]; however, evidence is limited and has been debated [1]. Our results provide further support for the characterization of *Sarcina* spp. by sporulation at alkaline pH and, in combination with our prior study [14], suggest that soil may serve as an environmental reservoir for these bacteria.

**Fig. 1.**
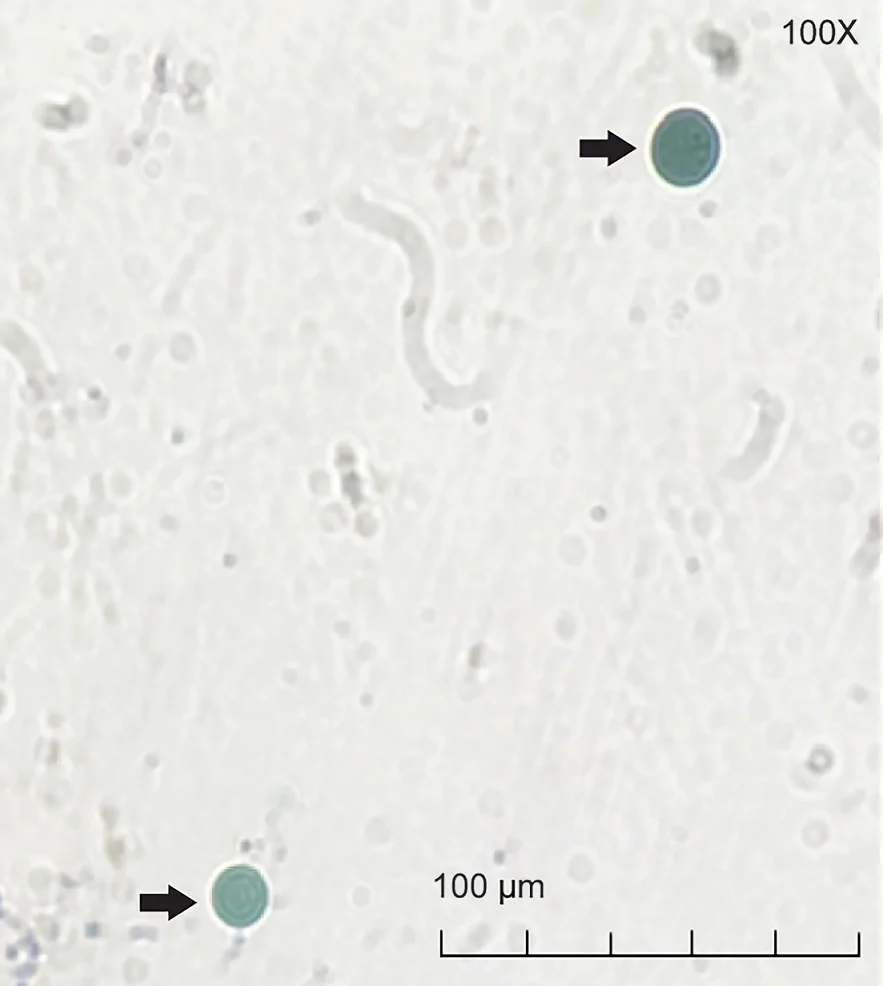
Strain JB3^T^ endospores (indicated by black arrows) produced under alkaline pH conditions *in vitro*. Photographed using a Panthera U microscope with a Moticam 5+ camera (Motic) and Motic Images Plus 3.0 (x64) software.

At pH 4, increased cell density of strain JB3^T^ was observed in the primary liquid culture over 24 h, but the organism did not survive serial dilutions and plating (data not shown). After 10 h, a 2-log increase in c.f.u. ml^−1^ of strain JB3^T^ was observed at pH 6 (Fig. 2). In contrast, at pH 8, an extended lag phase followed by a 2-log increase in c.f.u. ml^−1^ of strain JB3^T^ was observed by 24 h. A similar extended lag phase was observed at pH 9, but cell viability declined by 24 h. From these data, we can conclude that JB3^T^ has optimal *in vitro* growth at pH 6 in RC broth. Although S*arcina* have been shown to survive a wide pH range from 1 to 9.8 [1], the majority of evidence from growth at pH ≤2.2 come from environmental isolates [5] or abomasal and gastric contents [11]. Our results are similar to those reported by Crowther [6] in which culturing of *Sarcina* from human faecal samples at pH 4 was highly selective but also inhibited some strains.

**Fig. 2.**
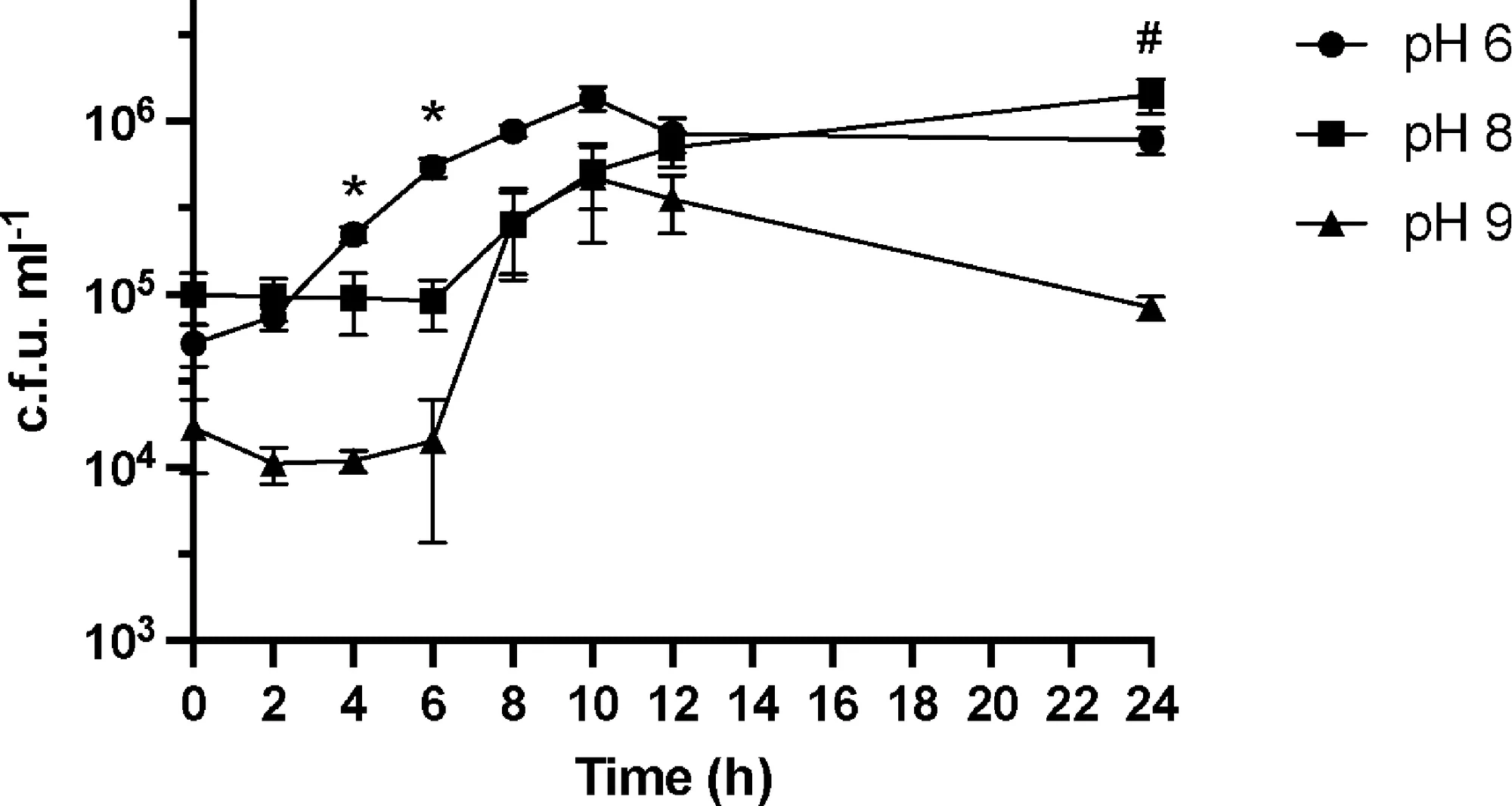
‘*Ca*. S. troglodytae’ strain JB3T growth curves at three pH values. Data points or bars represent the mean±sem of three independent experiments. Significant difference (*P*<0.05) between pH 6 and pH 9 (*) or pH 6 and pH 8 (#) as detected by Kruskal-Wallis test with post hoc Wilcoxon rank-sum tests and Benjamini–Hochberg adjustment for multiple comparisons. Figure generated in GraphPad Prism 10.

### Antibiotic susceptibility

Strain JB3^T^ was broadly susceptible to all antibiotics tested except for azithromycin and trimethoprim/sulfamethoxazole for which moderate susceptibility was observed and ceftriaxone for which resistance was observed (Table 2). These results are similar to the susceptibility profile of *S. ventriculi* isolates cultured from faeces of healthy zoo-housed monkeys in Europe, which demonstrated resistance to several cephalosporins [17].

**Table 2.** Susceptibility of strain JB3^T^ to antibiotics determined using the disc diffusion method The inhibition zones (diameter) were measured in millimetres, including the disc diameter (6 mm). Results are expressed as mean diameter±sd and interpreted as resistant <8 mm, moderately susceptible 8–21 mm and susceptible >21 mm.

Class	Antibiotic	Amount (µg)	Strain JB3^T^	*S. ventriculi* (ATCC 29069)
Macrolides	Azithromycin	15	19.7±0.6	20±0
	Clindamycin	2	30±0	33.3±0.6
Fluoroquinolones	Ciprofloxacin	5	22.7±1.5	25±0
	Enrofloxacin	5	25±1	30±0
Penicillins	Amoxicillin clavulanic acid	30	27.3±0.6	24.7±0.6
Tetracyclines	Doxycycline	30	38.3±0.6	39±0
Sulphonamides	Trimethoprim/sulfamethoxazole	25	11±1	10±0
Imidazoles	Metronidazole	5	24±2.6	21.7±0.6
Cephalosporins	Ceftriaxone	30	7.7±0.6	6.3±0.6

### Conclusion

This study is the first to describe viable *in vitro* growth conditions for ‘*Ca*. S. troglodytae’, a novel bacterium associated with ENGS, the leading cause of death for sanctuary chimpanzees in Sierra Leone since 2005. Further biochemical and physiological characterization, in combination with our prior studies [14, 15], provides additional support for the association between ‘*Ca*. S. troglodytae’ and ENGS through the identification of clinically relevant fermentation byproducts (i.e. gas and ethanol). Additionally, the identification of endospores in culture suggests that mitigation efforts may be challenging if the bacterium persists in an environmental reservoir. The culture methods described in this study will serve as the basis for future studies, namely, the development of a suitable animal model for infection studies to elucidate the role of ‘*Ca*. S. troglodytae’ in the aetiology of ENGS.

## Supplementary material

10.1099/acmi.0.001186.v4 Table S1.

## Abbreviations

ATCC: American Type Culture Collection
‘*Ca*. S. troglodytae’: ‘*Candidatus* Sarcina troglodytae’
c.f.u. ml^−1^: c.f.u. per millilitre
ENGS: epizootic neurologic and gastroenteric syndrome
RC: reinforced clostridial
TCS: Tacugama Chimpanzee Sanctuary
